# Protective effects of ginsenosides Rg1 and Rb1 against cognitive impairment induced by simulated microgravity in rats

**DOI:** 10.3389/fphar.2023.1167398

**Published:** 2023-04-24

**Authors:** Ning Jiang, Jingwei Lv, Yiwen Zhang, Xinran Sun, Caihong Yao, Qiong Wang, Qinghu He, Xinmin Liu

**Affiliations:** ^1^ Research Center for Pharmacology and Toxicology, Institute of Medicinal Plant Development (IMPLAD), Chinese Academy of Medical Sciences and Peking Union Medical College, Beijing, China; ^2^ Institute of Food Science and Technology, Chinese Academy of Agricultural Sciences (CAAS), Beijing, China; ^3^ Sino-Pakistan Center on Traditional Chinese Medicine, Hunan University of Medicine, Huaihua, China

**Keywords:** ginsenoside Rg1, ginsenoside Rb1, memory impairments, simulated weightlessness, rat

## Abstract

Microgravity experienced during space flight is known to exert several negative effects on the learning ability and memory of astronauts. Few effective strategies are currently available to counteract these effects. Rg1 and Rb1, the major steroidal components of ginseng, have shown potent neuroprotective effects with a high safety profile. The present study aimed to investigate the effects of Rg1 and Rb1 on simulated microgravity-induced learning and memory dysfunction and its underlying mechanism in the hindlimb suspension (HLS) rat model. Administration of Rg1 (30 and 60 μmol/kg) and Rb1 (30 and 60 μmol/kg) for 2 weeks resulted in a significant amelioration of impaired spatial and associative learning and memory caused by 4-week HLS exposure, measured using the Morris water maze and Reward operating conditioning reflex (ROCR) tests, respectively. Furthermore, Rg1 and Rb1 administration alleviated reactive oxygen species production and enhanced antioxidant enzyme activities in the prefrontal cortex (PFC). Rg1 and Rb1 also assisted in the recovery of mitochondrial complex I (NADH dehydrogenase) activities, increased the expression of Mfn2 and decreased the fission marker dynamin-related protein (Drp)-1expression. Additionally, Rg1 and Rb1 treatment increased the SYN, and PSD95 protein expressions and decreased the ratio of Bax:Bcl-2 and reduced the expression of cleaved caspase-3 and cytochrome C. Besides these, the BDNF-TrkB/PI3K-Akt pathway was also activated by Rg1 and Rb1 treatment. Altogether, Rg1 and Rb1 treatment attenuated cognitive deficits induced by HLS, mitigated mitochondrial dysfunction, attenuated oxidative stress, inhibited apoptosis, increased synaptic plasticity, and restored BDNF-TrkB/PI3K-Akt signaling.

## 1 Introduction

During long-term space trips, astronauts are exposed to peculiar and exceedingly complicated environmental conditions, of which microgravity is considered to be one of the main hazards to human health (Yoon et al., 2017; Nordeen and Martin, 2019). Actual (spaceflight) or simulated microgravity is known to induce a variety of physiological changes, especially in the central nervous system. In particular, it affects many aspects of brain function, including cognitive performance, posture control, locomotion, and manual control (Xiang et al., 2019). Hindlimb suspension (HLS) is the most commonly used small-animal model for simulating microgravity; it was developed in the 1980s and used to reproduce a cephalad blood and fluid shift, producing the same effects as microgravity in various organ systems such as the cardiovascular, immune, and nervous systems (Globus and Morey-Holton, 2016). The HLS model is widely used to assess the effects of microgravity on learning and memory deficits. Mounting evidence from various behavioral tasks (e.g., the Morris water maze, shuttle-box test, and object recognition test) has shown that exposure to simulated microgravity using the HLS model produces cognitive impairment resulting from increased levels of ROS, reduced BDNF expression, or alterations in neurotransmitter levels as well as impaired synaptic plasticity (Wang et al., 2016; Xiang et al., 2019).

**FIGURE 1:**
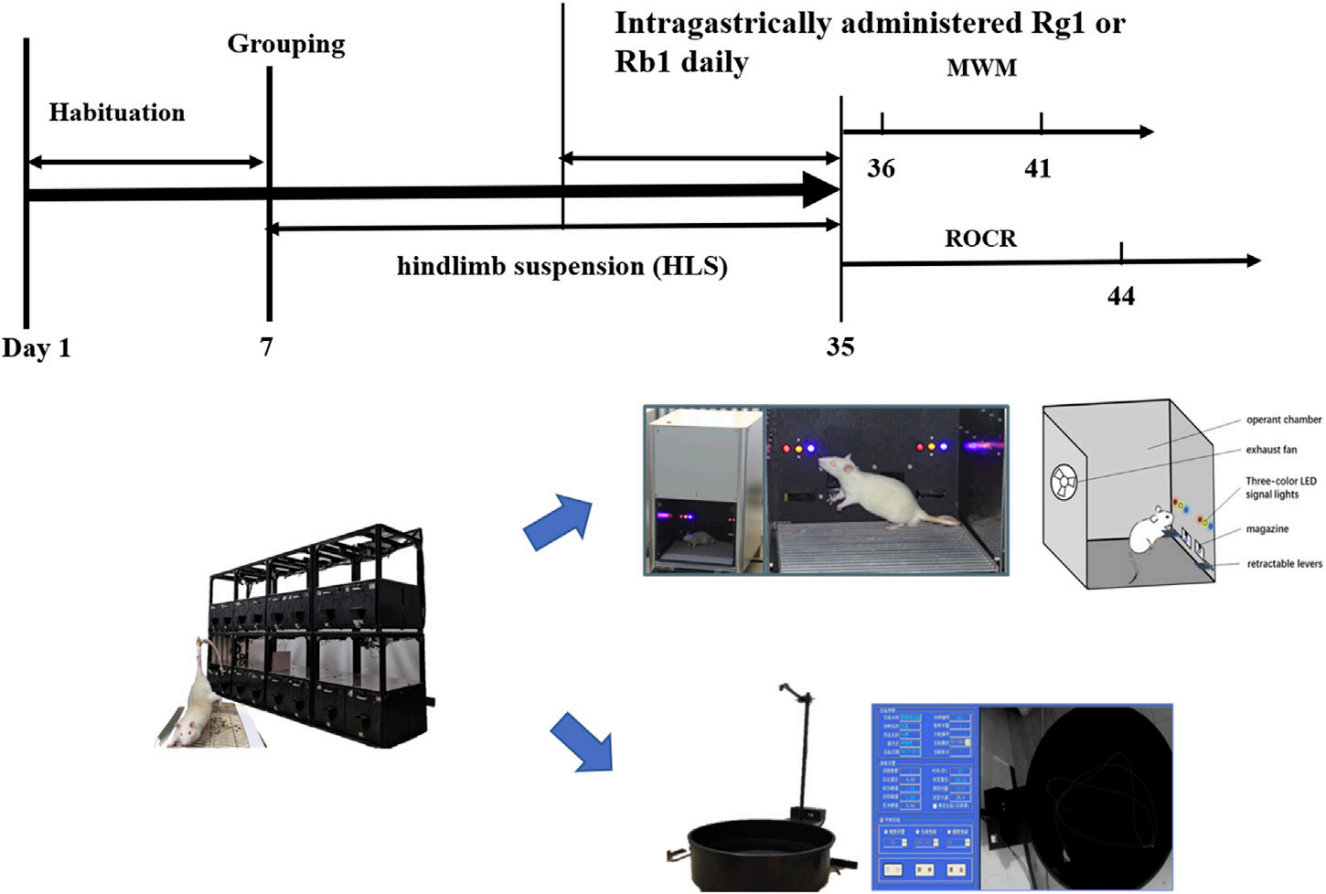
The experimental protocol of the study.

In recent years, herbal medicines have emerged as novel and attractive pharmacotherapeutic tools for the treatment of memory deficits, especially because of their effectiveness and high safety margins/profiles (Liu et al., 2016). Panax ginseng has gained immense popularity worldwide and its active ingredient is ginsenoside (Huang et al., 2015). Ginsenoside is known to exert multiple pharmacological effects on the neuronal, cardiovascular, and immune systems (Mohanan et al., 2018), especially in neurodegenerative diseases such as Alzheimer’s disease (AD), and effects on memory enhancement have gained significant attention in the past few years (Jiang et al., 2020). Rb1 (diol-type ginseng saponins) and Rg1 (triol-type ginseng saponins) are regarded as the main active components that are responsible for memory enhancement (Figure 1) (Wang et al., 2010) and recent studies have demonstrated the preventive and therapeutic benefits of ginsenosides Rb1 and Rg1 on cognitive deficits. Ginsenoside Rg1 has been shown to ameliorate hippocampal long-term potentiation (LTP) and memory by facilitating the clearance of AD-associated proteins and activation of the BDNF-TrkB pathway (Li et al., 2016). A previous study from our laboratory suggested that oral administration of ginsenosides Rb1 and Rg1 could mitigate cognitive impairment in senescence-accelerated (SAMP8) mice, reducing neuroinflammation, and ameliorate cognitive deficits in rats exposed to chronic restraint stress (Kezhu et al., 2017; Yang et al., 2020; Jiang et al., 2021). However, the effects of Rb1 and Rg1 on cognitive impairment induced by simulated microgravity are still unknown. Thus, in the present study, a rat model of HLS-induced cognitive dysfunction was employed to study the potential beneficial effects of ginsenosides Rb1 and Rg1 on the prevention of impairment of spatial, associative learning and memory, and the probable underlying mechanism.

## 2 Materials and methods

### 2.1 Chemicals and reagents

Ruifensi Biological Technology Co., Ltd. (Chengdu, China) provided Ginsenoside Rg1 (Rg1, purity>98%) and ginsenoside Rb1 (Rb1, purity>98%). Huperzine-A was purchased from Henan Tailong Biotech Co., Ltd. (Henan, China).

### 2.2 Animals

The Institute of the Chinese Academy of Medical Science Center, Beijing, donated male Wistar rats (*n* = 140, weight: 180–200 g). All rats were housed in standard conditions, including controlled humidity (55%) and temperature (20°C–22°C) and a 12-h:12-h light/dark cycle, with unrestricted access to water and food. The animal experiments were carried out with proper approval (Approval No. SYXK 2017-0020) following the requirements outlined by the Animal Research Committee of Peking Union Medical College’s Institute of Medicinal Plant Development (China).

### 2.3 Drugs and treatment

The rats were randomly assigned (*n* = 20 per group) to seven groups, namely, the non-HLS group (control), the HLS group, the HLS + huperzine-A (0.1 mg/kg), the HLS + Rg1 group (30 and 60 μmol/kg), and the HLS + Rb1 group (30 and 60 μmol/kg). On the 14th day of HLS administration, water, Rg1, Rb1, or Huperzine-A were administered intragastrically once daily until the complete behavioral assessment.

### 2.4 HLS model

HLS modeling was performed as previously described with some slight adjustments to recreate microgravity in space (Qiong et al., 2016; Lv et al., 2021). The rats were suspended in individual plastic cages with a 30-degree head-down tilt for 4 weeks. At the end of 4 weeks, all the above groups were subdivided into two groups (each with 10 rats) and subjected to behavioral tests such as the Morris water maze (MWM) and the reward operating conditioning reflex (ROCR) tests (Figure 1).

### 2.5 Behavioral tests

#### 2.5.1 Reward operating conditioning reflex test

##### 2.5.1.1 Food restriction

The rats’ food and water supplies were restricted until the reward training began. Sugar and water were used as a reward, and the body weights were slowly reduced to 80%–90% of the normal feeding weight over 10 days by controlling the amount of food and water given to the animals (Shi et al., 2013; Xu et al., 2017).

##### 2.5.1.2 Magazine training

During each training cycle, an unconditioned stimulus signal (blue signal light) was shown initially, followed by reinforcement with a reward substance. The cycle was repeated 30 times during the interval, including the conditioned stimulus and rewarding activities. In other words, the blue light signal was utilized as the stimulus, and the light was switched on periodically for 10 s at an interval of 30 s. The total number of times this step was repeated was 30, and the training time was 20 min daily. The reward gadget automatically provided a drop of 8% sucrose water as a reward substance when the blue light turned on. There was no sugar pump used after the cooldown period. This phase continued for 3 consecutive days.

##### 2.5.1.3 Lever-pressing reflex acquisition

During each training cycle, the animals pressed the left-side lever, resulting in an unconditioned stimulus that was later reinforced with a reward. The left pedal remained extended for 3 days after the rat had acquired the lever-pressing reflex. The blue signal light remained on for 10 s after the rats completed a left pedal action, activating a reward. The experiment was terminated after 30 min of training each day or 50 consecutive pedal presses during the training period.

#### 2.5.2 Morris water maze test

On day 28 of HLS treatment, the Morris water maze (MWM) test was used to investigate the effects of Rg1 and Rb1 on spatial memory (Morris, 1984; Xu et al., 2016). The water maze was a circular pool (180 cm in diameter, 40 cm high), and black ink was added to the water (23°C–25°C) to render it opaque. The only escape was an “invisible” platform (black-colored metal, 9 cm diameter) located 1.5 cm below the water level.

To learn the escape mechanism, each rat was tested for 2 trials/day for 5 consecutive days with each trial lasting 90 s. The rats spent 10 s on the platform before testing, and an animal was considered successful if it found the platform and stayed there for more than 2 seconds. The animal was led to the platform for 10 s after the test period (post-adaptation).

For the probe trial, the platform in the circular pool was removed 24 h after the escape acquisition test, and no pre-adaptation or post-adaptation was allowed in the experiment. The rats were placed in the diagonal quadrant of the pool, where the platform was situated, facing the wall from the center point of the pool wall, and they were given 90 s to swim and explore the pool. The number of target crossings served as a test of spatial memory.

### 2.6 Measurement of oxidative stress

After sacrifice of the rats, the prefrontal cortices were homogenized in 10 volumes of cold saline. The total protein content of the sample was determined using bovine serum albumin as the standard and a BCA Assay kit (Pierce, United States). The H_2_O_2_ contents, superoxide dismutase (SOD) activity, glutathione peroxidase (GSH-Px) activity, and malondialdehyde (MDA) concentration in the cortical tissue were measured using commercial assay kits, as directed by the manufacturer (Jiancheng, Nanjing, China) (Lu et al., 2020).

### 2.7 Western blotting

Western blotting was conducted as previously described with minor modifications (Shi et al., 2017). Proteins were separated on SDS-PAGE and transferred to nitrocellulose membranes (Millipore, Bedford, MA, United States). After blocking for 2 h in 5% nonfat milk in Tris-buffered saline with Tween-20 (TBST), the membranes were incubated overnight at 4°C with primary antibodies against Phospho-Akt (ab4060, 1:2000), BDNF (ab108319, 1:2000), Cytochrome C (ab133504, 1:5000), BAX (ab32503, 1:2000), Bcl-2 (ab194583, 1:1000), PSD95 (ab18258, 1:1000), TrkB (ab187041, 1:5000), Mitofusin 2 (ab133504, 1:5000) (all from Abcam Ltd., Cambridge, United Kingdom), and GAPDH (A19056, 1:1000; ABclonal Technology Co., Ltd., Wuhan, China). The membranes were then incubated for 1 h with a horseradish peroxidase-conjugated secondary antibody. An ECL Prime Kit was used to view the protein bands, and ImageJ 1.46r software (NIH, United States, RRID: SCR_003070) was used for quantification.

### 2.8 Histopathological examination

After the behavioral tests, the animals were anesthetized by pentobarbital sodium injection followed by transcardial perfusion with 4% paraformaldehyde in 0.01 M phosphate buffer for 24 h (4 rats in each group) after the last session. After harvesting, the tissues were embedded in paraffin and stained with H&E following standard procedures (Shi et al., 2019; Xie et al., 2019).

### 2.9 Statistical analysis

SPSS 22.0 was used for data analysis, and data are represented as the mean ± _standard error of the mean (SEM). We used either one-way ANOVA or repeated-measure two-way ANOVA to assess the differences between the mean values. All one-way ANOVAs were followed by *post hoc* analysis using the LSD test. All two-way ANOVAs were followed by Bonferroni *post hoc* analysis to examine isolated comparisons. *p* < 0.05 was regarded as statistically significant, and the outcome was expressed as the mean ± standard error of the mean (±SEM).

## 3 Results

### 3.1 Effects of ginsenosides Rg1 and Rb1 on learning and memory in HLS-exposed rats

**FIGURE 2:**
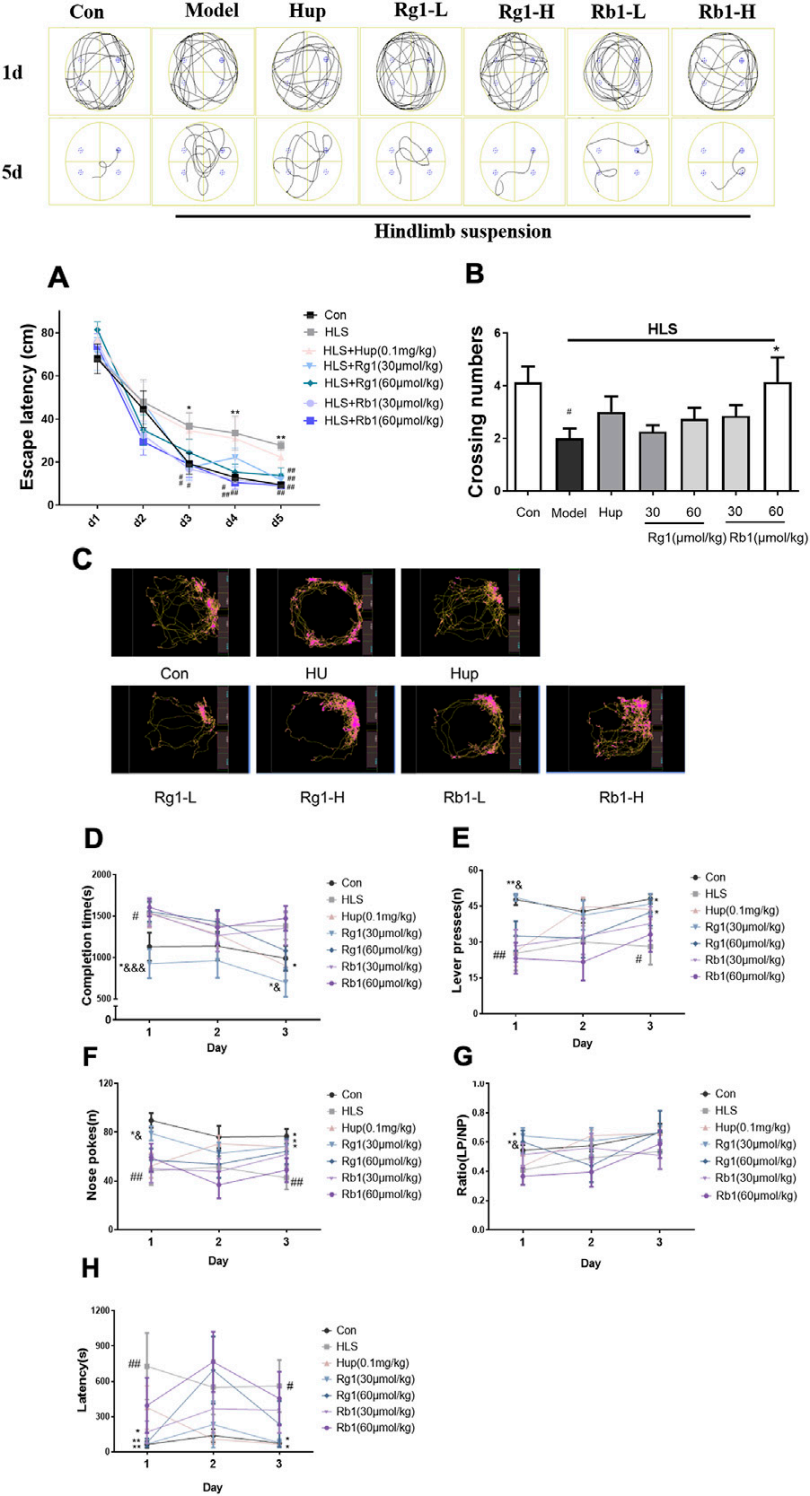
Ginsenosides Rg1 and Rb1 ameliorate cognitive dysfunction measured by behavioral tests in hindlimb suspension (HLS) model rats. **(A-B)** The Morris water maze test was used to measure escape latency and frequency of crossing the platform over 5 days. **(C–H)** Representation of moving path, Completion time, Lever presses (LPs), Nose-pokes (NPs), LP/NP ratio, and the latency on the acquisition of operant conditioning reflexes (lever-pressing) in the ROCR test. The data represent mean ± SEM; *N* = 10 rats per group. ^#^ *p* < 0.05, ^##^ *p* < 0.01, significantly different from control; ^*^ *p* < 0.05, ^**^ *p* < 0.01, significantly different from HLS group. ^&^ *p* < 0.05, ^&&^ *p* < 0.01, ^&&&^ *p* < 0.001, significantly different from Rb1 group.

As shown in Figure 2A, in the MWM acquisition trial, the latency to reach the platform was significantly longer in the HLS group than in the CON group from days 3–5 (*p* < 0.05, *p* < 0.01, *p* < 0.01, respectively). However, treatment with Rb1 (30 and 60 μmol/kg) reduced the escape latency on days 3–5 when compared with the HLS model rats (*p* < 0.05). Administration of Rg1 (30 and 60 μmol/kg) also markedly improved HLS-induced increments in the escape latency from day 4. Moreover, the rats in the HLS group took shorter times to pass over the hidden platform than those in the control group in the probe test (Figure 2B, *p* < 0.05). These times were further reduced after treatment with high-dose Rb1 (60 μmol/kg).

On day 1 of the ROCR test (Figures 2C,D), the completion time in the model group was considerably longer than that in the control group (*p* < 0.05). Rg1 (30 μmol/kg) treatment led to significantly reduced completion times on days 1 and 3 (*p* < 0.05). On days 1 and 3 (*p* < 0.05), Rg1 considerably improved the completion time compared to the same dose of Rb1. On days 1 and 3, the number of lever presses (LPs) in the untreated model group was progressively reduced compared with the control group (Figure 2E, *p* < 0.05) while treatment with Rg1 (30 μmol/kg) raised the LP number considerably (*p* < 0.05). The number of nose-pokes (NPs) in the HLS rats was fewer compared with the control rats from day 1 to day 3 (Figure 2F, *p* < 0.05). Nevertheless, both Rg1 (30 μmol/kg) and huperzine A treatments significantly increased the NP numbers in HLS rats from day 1 to day 3. The latency of the model group was significantly increased on day 1 and day 3 (Figure 2H, *p* < 0.05). Rg1 (30 and 60 μmol/kg) treatment significantly shortened the latency on day 1 (*p* < 0.01). The latency of the Rg1 (30 μmol/kg) group on day 3 and the Rb1 low-dose group on day 1 was significantly shortened (*p* < 0.05). Furthermore, rats treated with Rg1 (30 and 60 μmol/kg) exhibited significant improvements in the LP/NP ratio on day 1, and LP/NP ratio for the low-dose Rg1 (30 μmol/kg) group was observed to be higher compared with the low-dose Rb1 group (Figure 2G, *p* < 0.05).

### 3.2 Effects of ginsenosides Rg1 and Rb1 on the markers of oxidative stress in the prefrontal cortex

**FIGURE 3:**
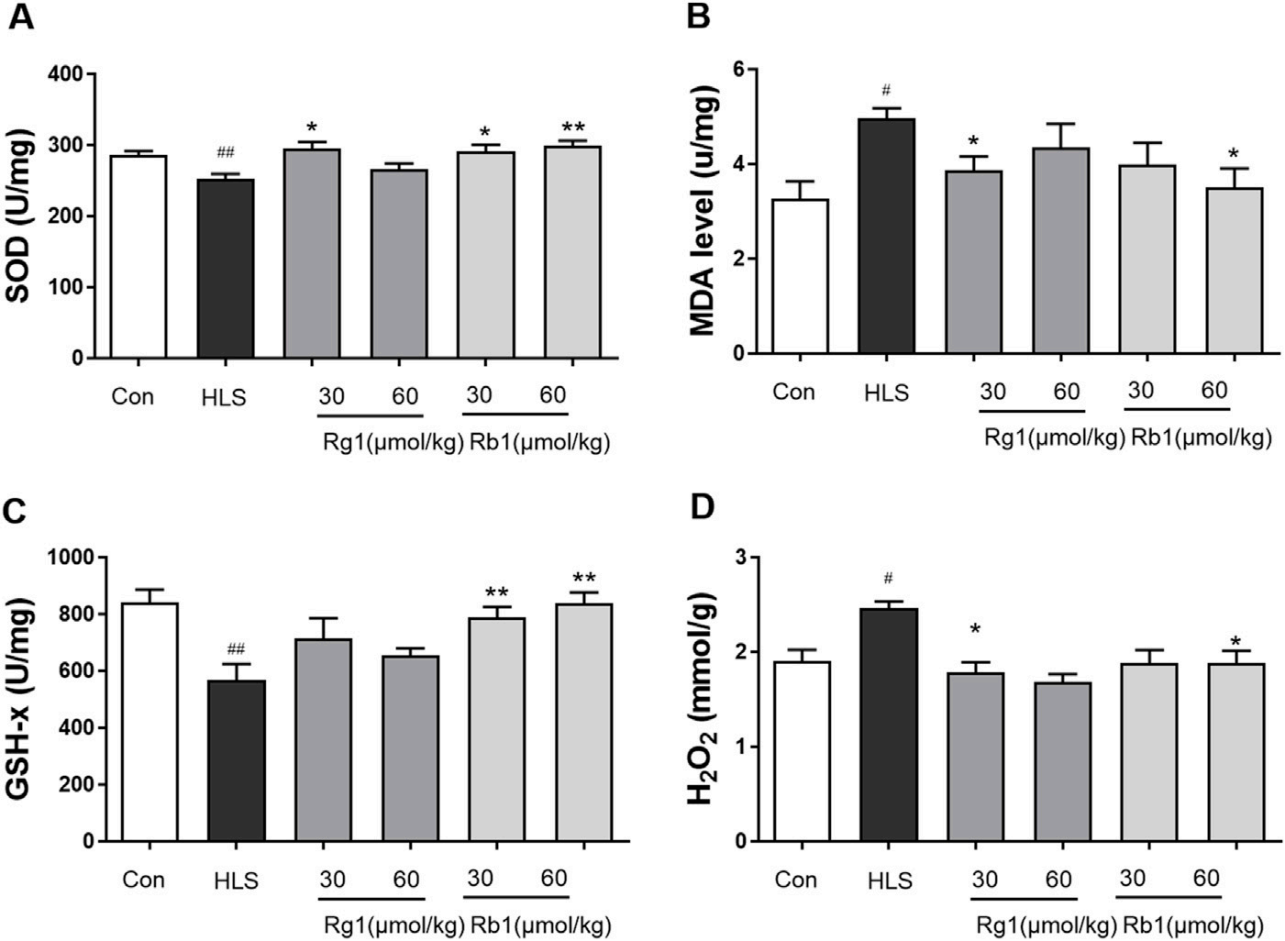
The effect of ginsenosides Rg1 and Rb1 on oxidative stress markers in hindlimb suspension (HLS) rats. **(A)** SOD activity, **(B)** MDA level, **(C)** GSH-Px activity, and **(D)** H_2_O_2_ contents in the prefrontal cortex. Data represent means ± SEM; *N* = 6 rats per group. ^#^ *p* < 0.05, ^##^ *p* < 0.01, significantly different from control; ^*^ *p* < 0.05, ^**^ *p* < 0.01, significantly different from HLS group.

The activities of SOD and GSH-Px in the prefrontal cortices (PFCs) of the HLS group were considerably lower in comparison with those in the control group [SOD, F (5, 30) = 4.366, *p* < 0.01; GSH-Px, F (5, 30) = 4.792, *p* < 0.01], as shown in Figures 3A, C. Rg1 (30 μmol/kg) and Rb1 (30 and 60 μmol/kg) treatment, on the other hand, led to a marked increase in SOD activity. In contrast, Rb1 (30 and 60 mol/kg) treatment significantly ameliorated the depleted GSH-Px levels in the PFC (*p* < 0.05). MDA and H_2_O_2_ levels in the PFC of rats exposed to HLS increased significantly [H_2_O_2_, Figure 3B, F (5, 30) = 3.097, *p* < 0.01; Figure 3D, MDA, F (5, 30) = 2.175, *p* < 0.01]. Treatment with Rg1 (60 μmol/kg) and Rb1 (60 μmol/kg) significantly reduced this increment (*p* < 0.05.).

### 3.3 Influence of ginsenosides Rg1 and Rb1 on the expression of mitochondrial OXPHOS, Drp1, and Mfn2 proteins in the prefrontal cortex

**FIGURE 4:**
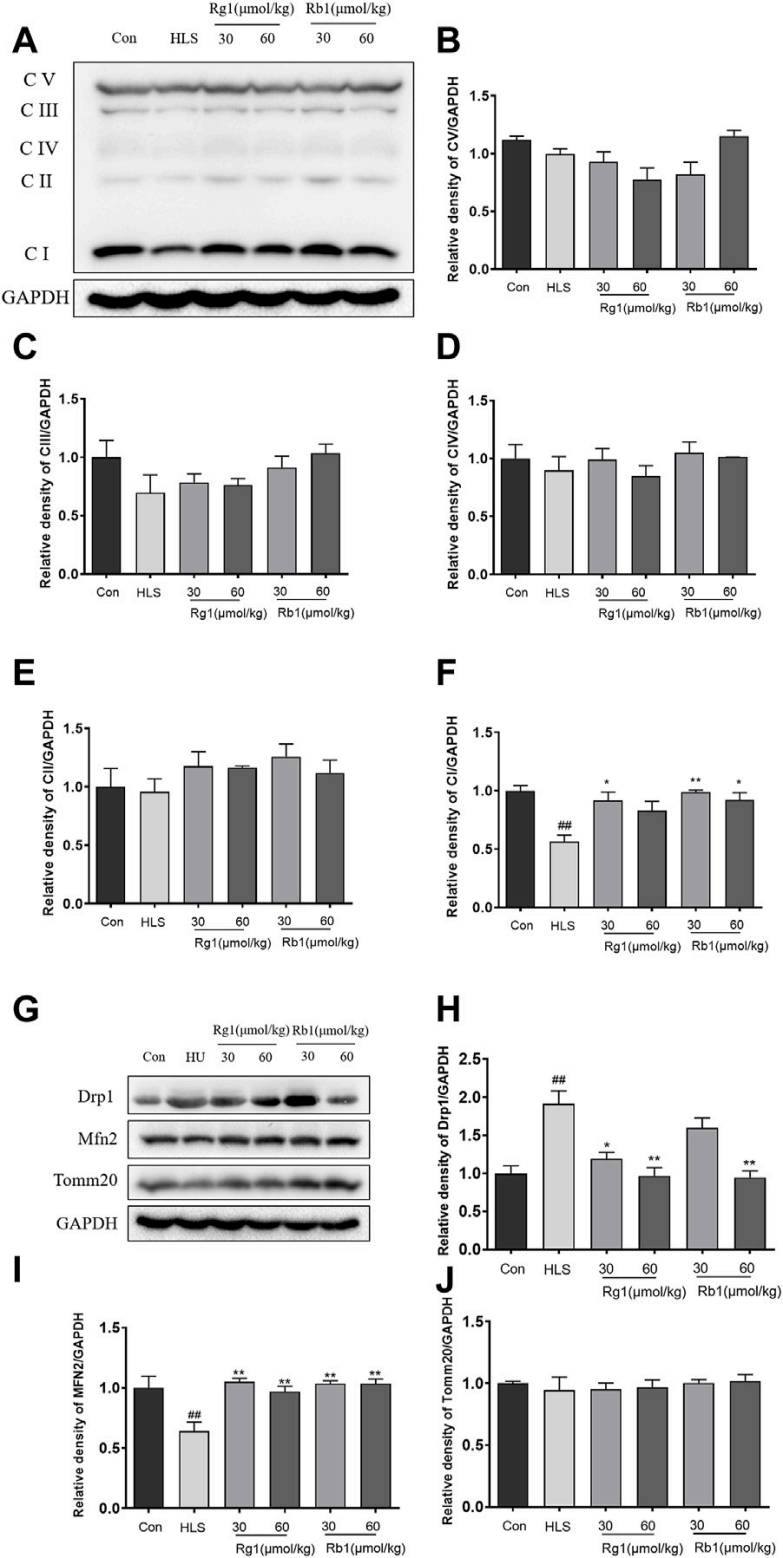
The effects of ginsenosides Rg1 and Rb1 on mitochondrial function of hindlimb suspension (HLS) rats. **(A–F)** Protein bands and relative expression levels of CV/GAPDH, CIII/GAPDH, CIV/GAPDH, C-II/GAPDH, and CV/GAPDH in the prefrontal cortex. **(G–J)** Protein bands and relative expression levels of MFN2 and Drp1. Data represent means ± SEM; *N* = 4 rats per group. ^#^ *p* < 0.05, ^##^ *p* < 0.01, significantly different from control; ^*^ *p* < 0.05, ^**^ *p* < 0.01, significantly different from HLS group.

As shown in Figures 4A–E, there were no significant differences between the groups in the levels of C-V, C-III, C-IV, and C-II in the PFCs. However, the contents of C -I in the PFC of the HLS model group were significantly decreased [Figure 4F, F (5, 23) = 6.658, *p* < 0.01]. Treatment with Rg1 (30 μmol/kg) and Rb1 (30 and 60 μmol/kg), on the other hand, resulted in a substantial increase in C-I (*p* < 0.05). The HLS group had significantly higher Drp1 protein levels in the mitochondrial division [Figure 4H, F (5, 23) = 5.170, *p* < 0.01], whereas Rg1 and Rb1 treatment blocked the enhanced expression of Drp1 (*p* < 0.05). Compared to the control group, reduce expression of the fusion protein Mfn2 was observed in the PFCs of the HLS model group [Figure 4I; F (5, 23) = 6.364 *p* < 0.01]. The administration of both ginsenosides increased the expression of Mfn2 (all *p* < 0.01). The expressions of Tomm 20 were no significant difference among all groups (Figure 4J).

### 3.4 Influence of ginsenosides Rg1 and Rb1 on the expression of BDNF, p-Akt/AKT, TrkB, SYN, and PSD-95 in the prefrontal cortex

**FIGURE 5:**
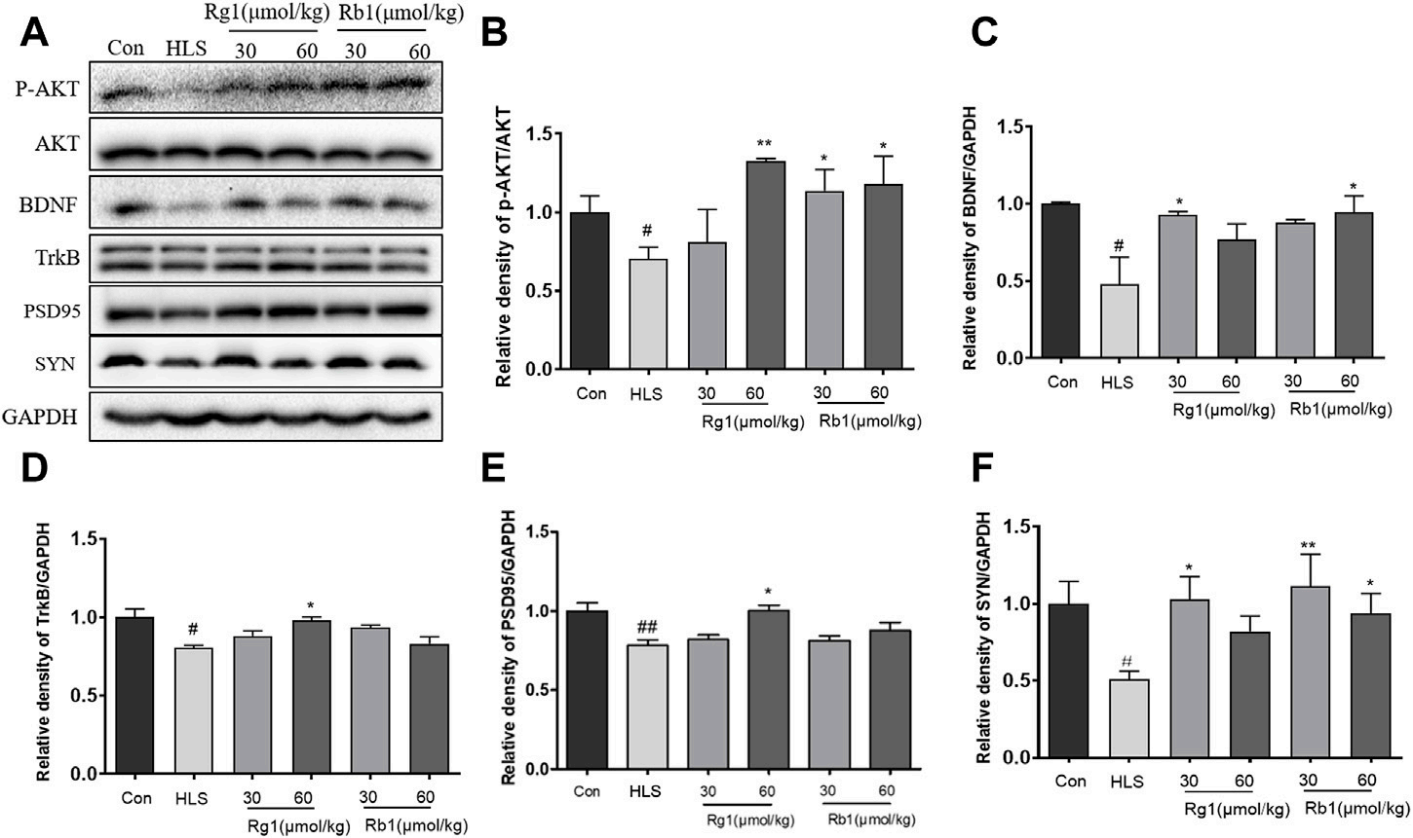
Ginsenosides Rg1 and Rb1 influence the expression of BDNF signaling pathway-related proteins in hindlimb suspension (HLS) rats. **(A–F)** Protein bands and relative expression levels of p- Akt/AKT, TrkB, BDNF, PSD-95, and SYN in the PFC. Data represent means ± SEM; *N* = 4 rats per group. ^#^ *p* < 0.05, ^##^ *p* < 0.01, significantly different from control; ^*^ *p* < 0.05, ^**^ *p* < 0.01, significantly different from HLS group.

As shown in Figures 5A–D, the levels of p-Akt/AKT [F (5, 23) = 3.171, *p* < 0.01], TrkB [F (5, 23) = 2.701, *p* < 0.01], and BDNF [F (5, 23) = 5.439, *p* < 0.01] were markedly decreased in the HLS model group compared to the control group. Rg1 (30 μmol/kg) and Rb1 (30 μmol/kg and 60 μmol/kg) significantly increased the levels of p-Akt/AKT (*p* < 0.01, *p* < 0.05, respectively). Rg1 (30 μmol/kg) and Rb1 (60 μmol/kg) treatments significantly reversed the expression trend of BDNF (*p* < 0.05). Furthermore, Rg1 (60 μmol/kg) treatment significantly increased the TrkB level in model animals (*p* < 0.05). The expression of both SYN [Figure 5F, F (5, 23) = 3.743, *p* < 0.01] and PSD-95 [Figure 5E, F (5, 23) = 7.322, *p* < 0.05] were found to be substantially decreased in the PFCs of HLS rats compared to the control rats. Additionally, Rg1 (60 μmol/kg) treatment resulted in a substantial increment in the expression of PSD-95 and SYN, while Rb1 (30 and 60 μmol/kg) significantly reversed the observed decrease in the expression of SYN (*p* < 0.05).

### 3.5 Effects of ginsenosides Rg1 and Rb1 on apoptosis, apoptosis-associated proteins, and neuronal cell loss in the prefrontal cortex

**FIGURE 6:**
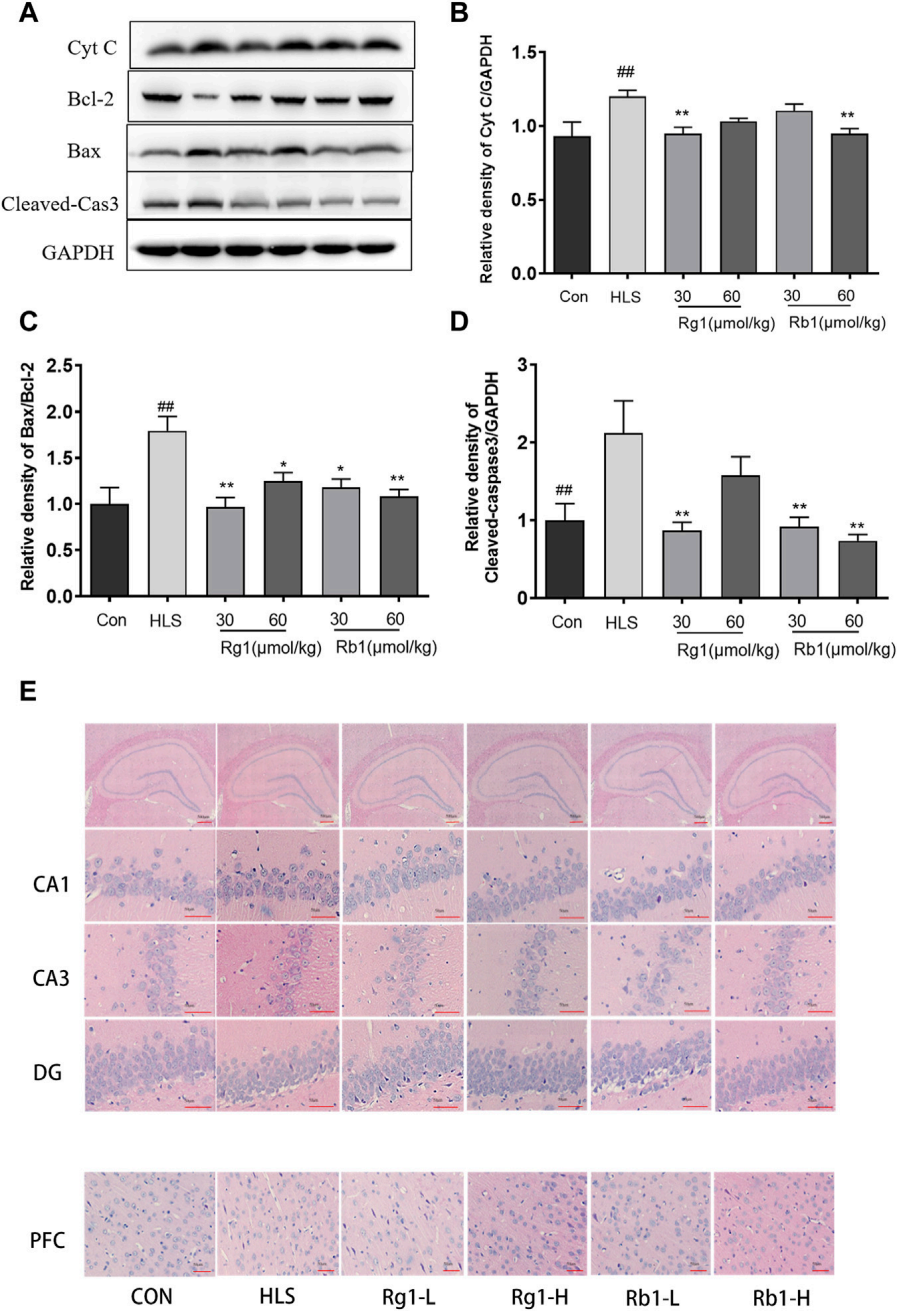
The effects of ginsenosides Rg1 and Rb1 on apoptosis, levels of apoptosis-associated proteins, and neuronal cell loss in hindlimb suspension (HLS) rats. **(A–D)** Protein bands and relative expression levels of Cyt C, Bcl-2/Bax, and cleaved-caspase 3 in the PFC. **(E)** The HLS rat hippocampal CA1, CA3, DG, and PFC regions were stained with hematoxylin-eosin (at ×400 magnification power). Data represent means ± SEM; *N* = 4 rats per group. ^#^ *p* < 0.05, ^##^ *p* < 0.01, significantly different from control; ^*^ *p* < 0.05, ^**^ *p* < 0.01, significantly different from HLS group.

The levels of Cyt C [F (5, 23) = 4.459, *p* < 0.01], cleaved-caspase 3 [F (5, 23) = 3.268, *p* < 0.01], and the Bax/Bcl-2 ratio [F (5, 23) = 2.310 *p* < 0.01], as shown in Figures 6A–D, were substantially increased in the PFCs of the HLS model group (*p* < 0.01). However, treatment with Rg1 and Rb1 significantly reduced these levels (*p* < 0.05).

Marked morphological changes were observed in both the PFC and hippocampus of HLS rats (Figure 6E), with neurons showing a loose organization and uneven cytoplasmic distribution. Neuronal cells with large nuclei were seen in the hippocampi and PFCs of rats treated with Rg1 and Rb1. These neurons adopted an ordered arrangement, suggesting that Rg1 and Rb1 treatment could notably reduce neuronal cell loss.

## 4 Discussion

Investigation of the effects of long-term treatment with the ginsenosides Rg1 and Rb1 on impaired spatial and associative learning and memory induced by HLS using the MWM test and reward-directed instrumental conditioning task showed that treatment with these ginsenosides led to dramatic improvements. Moreover, Rg1 and Rb1 treatment dramatically attenuated mitochondrial damage, reduced ROS production, inhibited neural cell apoptosis, and activated the BDNF-TrKB/PI3K-Akt signaling pathway in the PFCs of HLS model rats.

Deep space exploration poses risk to neural or tissue damage, cognitive function impairments, behavioral changes, and motor deficits. A recent study longitudinally compared a pair of monozygotic twin astronauts for 340-days, where one of the twins experienced the spaceflight environment on ISS, and simultaneously the other twin endures Earth environment (Mhatre et al., 2022). The results indicatesspaceflight specific effects such as altered circulating immune cytokines and metabolites levels, changes in telomere length, gene regulation at epigenetic as well as transcriptional levels, DNA damage, microbiome alterations, and attenuated cognitive performance (Garrett-Bakelman et al., 2019). In the present study, tail suspension was used (with animals angled 30° head-down), as it is the internationally recognized means of simulating weightlessness, and two types of cognitive-behavioral tests, each representing a different form of cognitive function, were conducted. Firstly, the MWM test was conducted to assess long-term spatial memory (Lu et al., 2018). Our results showed that HLS exposure generated severe neurocognitive deficits in spatial learning and memory, as indicated by the increase in escape latency during the acquisition phase and the decrease in numbers of crossings during the probing session (Zhang et al., 2018). Rg1 and Rb1 treatments enhanced MWM performance over that of the HLS model group, indicating that these ginsenosides improved spatial memory. The study found that Rb1 improved escape latency in the acquisition phase faster than Rg1 and that Rb1 therapy increased the number of crossings in the probing test. After 2 weeks of Rb1 and Rg1 treatment, Rb1 was found to have improved spatial memory more than Rg1 in HLS model rats. ROCR is a vital tool for identifying behavioral adaptation, as it allows for fast behavioral changes in response to changing conditions, offering a survival advantage (Dayan and Balleine, 2002; Shi et al., 2013). By pressing a lever, the animal receives a reward while performing the activity, making learning fun and reinforcing the activity. Two systems control instrumental behavior: the goal-directed process and the stimulus-response (S-R) habit mechanism (Balleine and Dickinson, 1998). In the current work, rats in the Rb1 and Rg1 low-dose groups could complete the task in the LP training course after 4 weeks of HLS treatment, while the former took longer. However, the other groups could not complete the task on the first day after modeling. The completion time and operation latency were higher in the model group compared to the control group, while the LP and NP values and the LP/NP ratio were decreased. These findings suggested that model rats’ exploration times, executive ability, and lever pressing efficiency were reduced, and model rats could not focus on the fixed ratio response test, necessitating a longer time to adjust even to a familiar setting. Rb1 administration reduced these operational delays and improved the focus of the animals, whereas Rg1 administration reversed the changes in the five indices described above and increased the interest in exploration, attentiveness, and operational ability. A significant difference was observed in comparison with the same dose of Rb1, indicating a better improvement effect. The findings of these two behavioral tests provided evidence for Rb1 and Rg1-mediated amelioration of HLS-induced learning and memory impairment. Thus, this treatment not only resulted in a significant improvement in spatial memory but also in associative learning and memory. This manuscript is the first study of ginsenosides for cognitive impairment induced by simulated microgravity.

Chronic stress-induced memory loss has been linked to oxidative stress damage. The accumulation of excess ROS overpowers the body’s antioxidant defense system, causing permanent damage to membrane lipids, proteins, and nucleic acids, intracellular damage accumulation, cognitive impairment, and cell death (Majdi et al., 2016; Ballard and Towarnicki, 2020). SOD, CAT, and GSH-Px are antioxidant enzymes that protect antioxidant systems. A recent study linked HLS-induced memory loss to increased oxidative stress (Wang et al., 2021). In agreement with these findings, HLS increased oxidative stress measured by MDA and H_2_O_2_ levels and decreased SOD and GSH-Px activity in the PFC. The ability of Rg1 and Rb1 to scavenge oxygen free radicals and thus enhance antioxidant functions may contribute to their protective effects against HLS-induced cognitive loss.

Mitochondrial dysfunction is a widely accepted primary cause for the development of cognitive impairment, particularly the pathogenesis of most neurodegenerative disorders, such as AD (Li J. et al., 2019). The primary purpose of mitochondria, in conjunction with cellular respiration, is to produce ATP through oxidative phosphorylation. These systems include the NADH succinic acid, CoQ reductase (complex I), and the mitochondrial respiratory chain composed of five enzyme complexes, namely, CoQ reductase (complex II), CoQcytochrome C reductase (complex III), cytochrome C oxidase (complex IV), and ATP synthase (complex V) (Stock et al., 2000). The mitochondrial complex I is the most critical of the five enzyme complexes in the respiratory chain, affecting both the respiratory chain and ATP anabolism. The Drp1 protein possesses GTPase activity and promotes fission by a chain formation in the mitochondrial outer membrane, which further promotes mitochondrial fission. OPA-1, Mfn1, and Mfn2, on the other hand, mediate fusion (Chan, 2020). In the current study, HLS exposure resulted in a decrease in complex I activity and Mfn2 protein levels, and an increase in the Drp1 protein expression in the rat PFCs. However, long-term Rg1 and Rb1 treatment reversed this, suggesting that Rg1 and Rb1 significantly improved mitochondrial function in HLS-exposed mice.

According to earlier research, aberrant mitochondrial dynamics influence apoptosis, and the mitotic protein Drp1 promotes Bax oligomerization and plays a role in apoptosis regulation. Overexpression of the fusion proteins Mfn2 and OPA1 reduces Bax activation while Mfn1 and Mfn2 promote sensitivity to apoptosis in cells (Camperchioli et al., 2011). Cytochrome C is an essential factor in mitochondria-mediated apoptotic pathways. The release of cytochrome C is an important indicator of mitochondrial damage. Bax and Bcl2 are members of the Bcl2 family and regulate mitochondrial cytochrome C release, caspase 3 activation, and apoptosis (Fuentealba et al., 2009; Jiang et al., 2019a). Several studies have shown that increased apoptosis occurs in various mammalian cell types in space and simulated microgravity (SMG) (Lewis et al., 1998; Kumari et al., 2009). In agreement with these findings, HLS exposure was found to enhance the Bax/Bcl2 ratio and the expression of cleaved caspase 3 and cytochrome C. Long-term administration of Rg1 and Rb1 in HLS rats, on the other hand, significantly reversed these effects, preventing neuronal death. Considering all of the evidence, Rg1 and Rb1’s neuroprotective effects in HLS rats may be partially a result of their capacity to inhibit apoptosis.

Mitochondrial insufficiency also leads to changes in synaptic density, which in turn results in significant impairment of cognitive function (D'Souza et al., 2015). SYN and PSD-95 are two key indicators and regulators of dynamic synaptic plasticity (Janz et al., 1999). Previous studies have reported that HLS exposure induced cognitive dysfunction and synaptic plasticity deficits in mice (Xiang et al., 2019). In agreement, our data showed that the expression of SYN and PSD-95 underwent a significant decline in the HLS model group, which was reversed by treatment with Rb1 and Rg1, indicating that both ginsenosides may enhance synaptic plasticity in the HLS model. In addition, a considerable amount of evidence suggests that a reduction in the level of BDNF in the PFC (presumably including the hippocampus) is strongly linked to cognitive impairment induced by simulated microgravity (Wu et al., 2017; Zhai et al., 2020). BDNF is a nutritional factor, which is known to play a major role in synaptic plasticity and neuronal stress resistance (Zhang et al., 2021). It is closely linked to various aspects of learning and memory processing (Yamada et al., 2002). Tyrosine kinase receptor B (TrkB) is a high-affinity receptor for BDNF and can trigger downstream intracellular PI3K/Akt signaling. The PI3K/Akt pathway is strongly implicated in the promotion of neuronal survival. It is also known to confer protection against apoptosis (Jiang et al., 2019b). BDNF has been previously reported to regulate the scaffolding protein PSD-95 via PI3K/Akt signaling (Li M. et al., 2019).

In the present study, HLS exposure significantly reduced the phosphorylation of TrkB, BDNF, and AKT in the PFC, together with reduced SYN expression. However, long-term administration of Rg1 and Rb1 to HLS-exposed rats significantly reversed these effects. This finding also raises the possibility that Rg1 and Rb1 treatment may affect BDNF-PI3K/Akt signaling in the PFC, which controls the expression of SYN and PSD95 and thus synaptic plasticity, thus reducing cognitive deficits in the rats.

## 5 Conclusion

In conclusion, the present study is the first to demonstrate that treatment with ginsenosides Rg1 and Rb1 protected against HLS-induced memory impairment. The underlying mechanism responsible for this protective influence may originate from its mitochondria-targeted antioxidant activity and regulation of the BDNF-TrkB/PI3K-Akt signaling axis, involving inhibition of apoptosis and an increase in synaptic plasticity. The present study highlights the use of ginsenosides Rg1 and Rb1 as novel candidate agents to counteract cognitive dysfunction induced by long-term spaceflight.

## Data Availability

The original contributions presented in the study are included in the article/Supplementary Materials, further inquiries can be directed to the corresponding author.

## Abbreviations

HLS; hindlimb suspension, MWM; Morris water maze test, ROCR; Reward operating conditioning reflex test, PFC; prefrontal cortex, LTP; long-term potentiation, Rb1; ginsenoside Rb1, Rg1; ginsenoside Rg1, LPs; lever presses, NPs; nose-poke activities, SOD; superoxide dismutase, MDA; Malondialdehyde, GSH-x; glutathione peroxidase, H_2_O_2_; Hydrogen peroxide, Drp1; dynamin-related protein 1, Mfn2; mitofusin-2, BDNF; Brain-derived neurotrophic factor, PSD95; postsynaptic density, SYN; synaptophysin, Cyt C; Cytochrome C.
