# Supplementary material for: Protective effects of ginsenosides Rg1 and Rb1 against cognitive impairment induced by simulated microgravity in rats
Source: Front Pharmacol. 2023 Apr 24;14:1167398. doi: 10.3389/fphar.2023.1167398 (PMC10164943; doi:10.3389/fphar.2023.1167398)
Supplement: Supplementary file 1 [file DataSheet1.ZIP › original data/WB(1).pptx]

## Slide 1
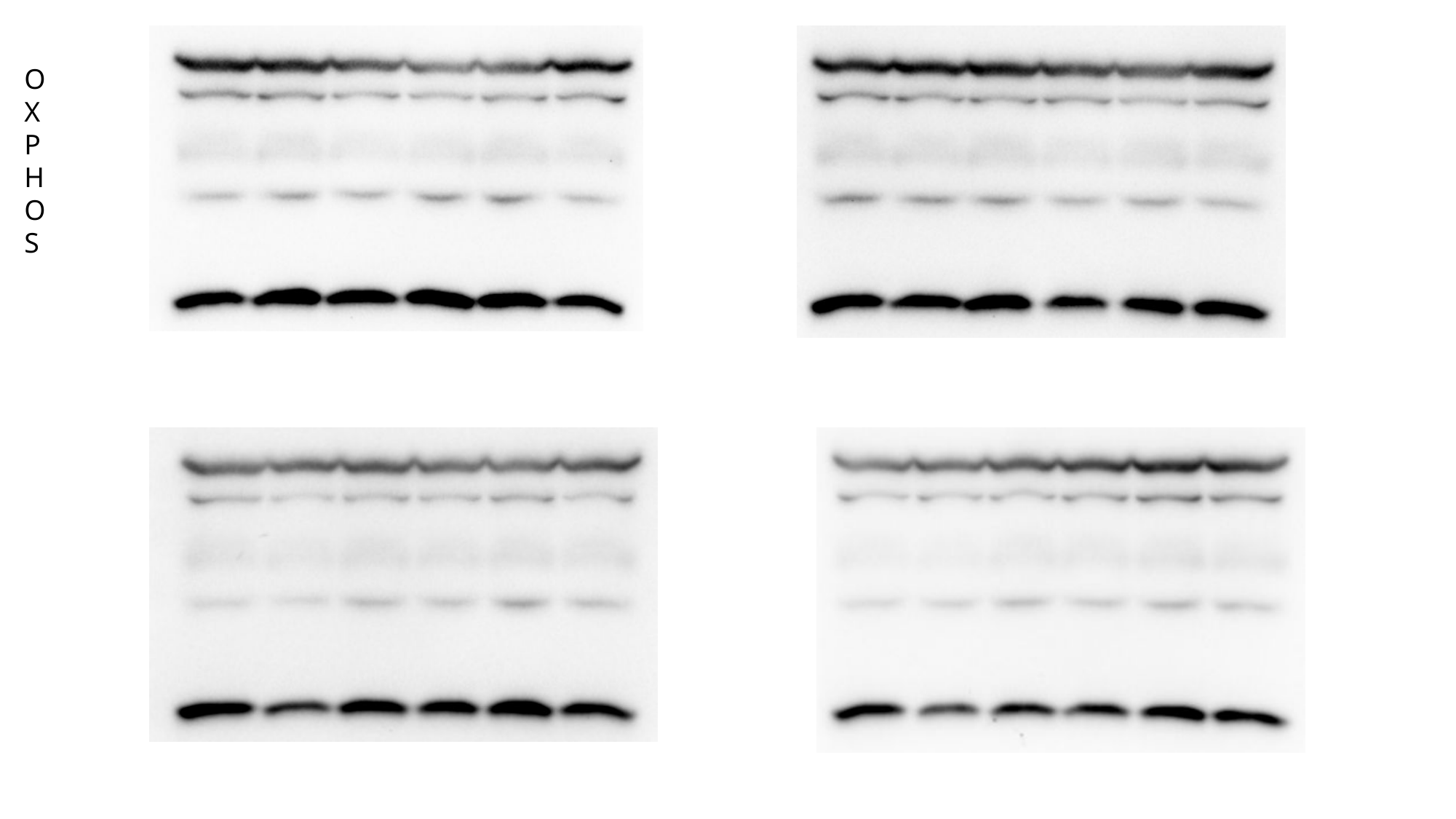

OXPHOS

## Slide 2
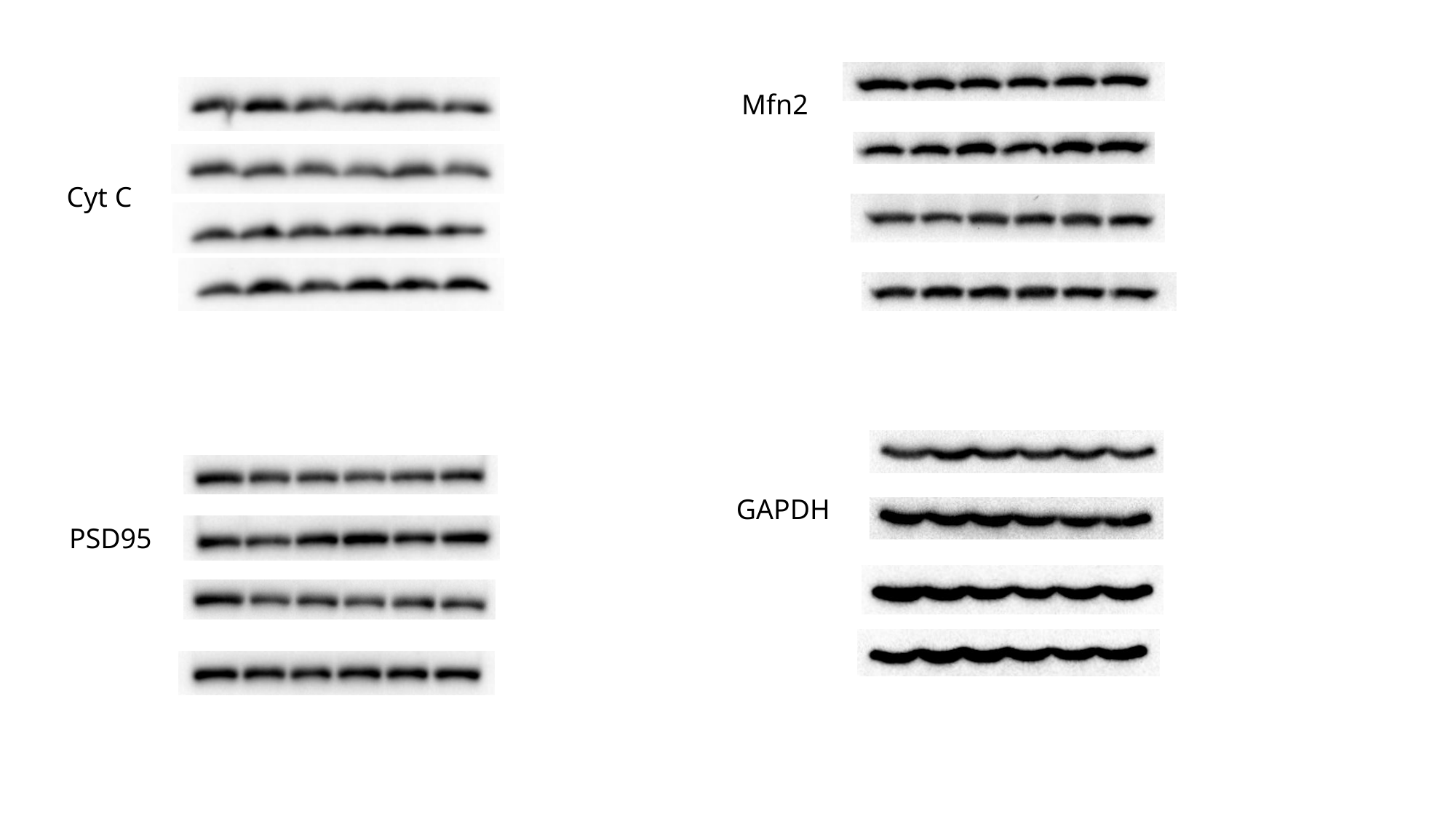

Mfn2
Cyt C
GAPDH
PSD95

## Slide 3
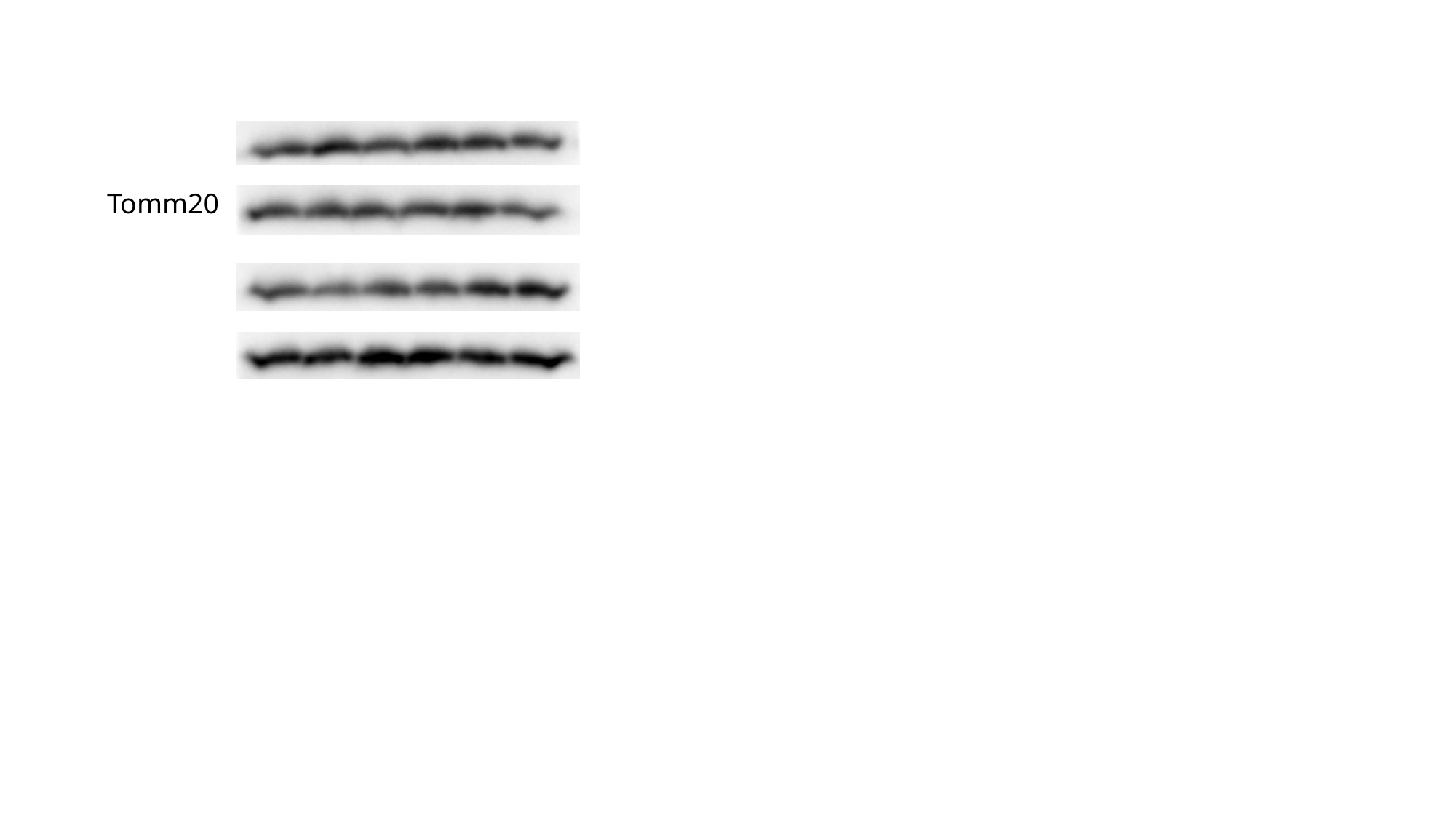

Tomm20

## Slide 4
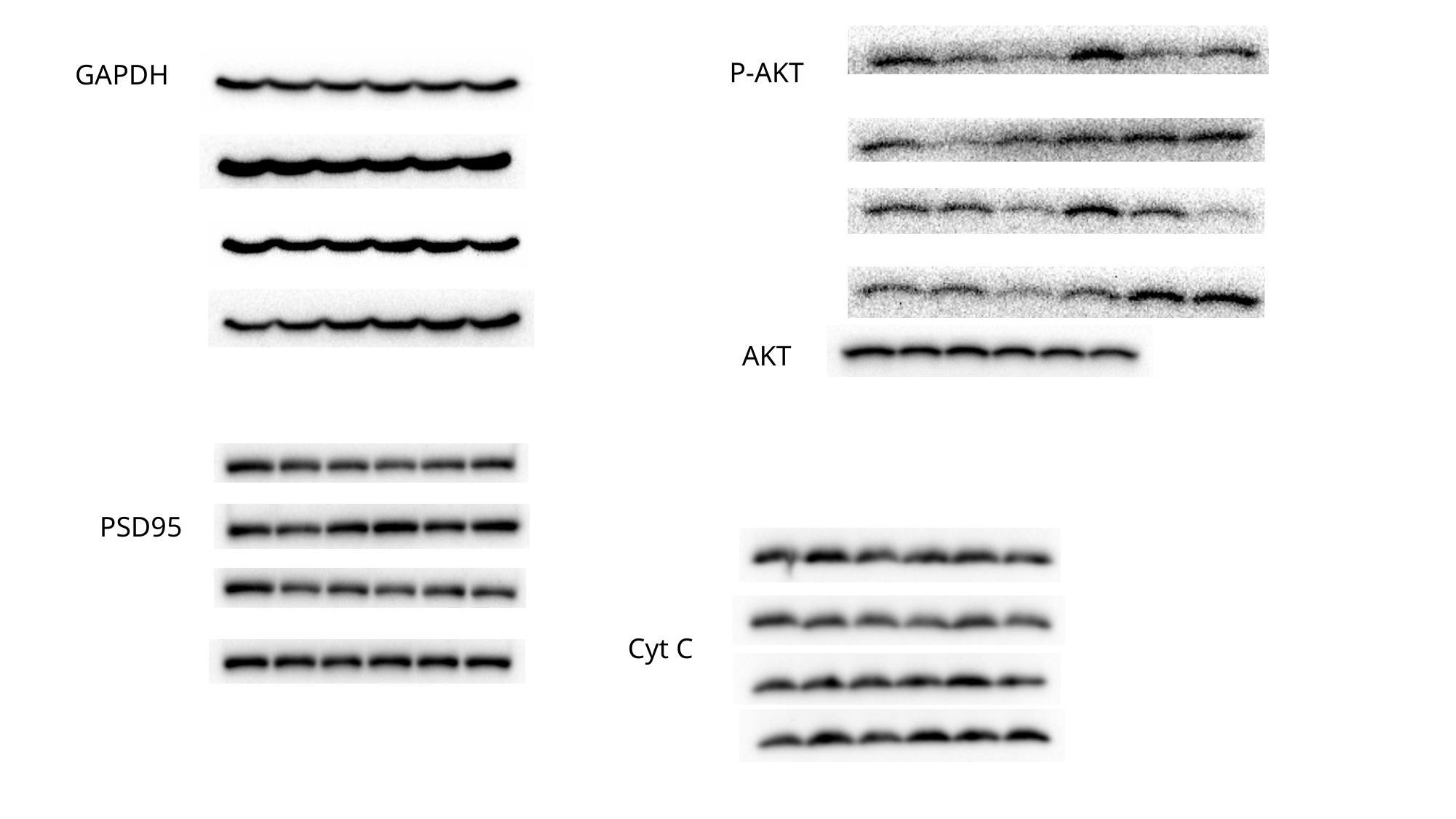

P-AKT
GAPDH
AKT
PSD95
Cyt C

## Slide 5
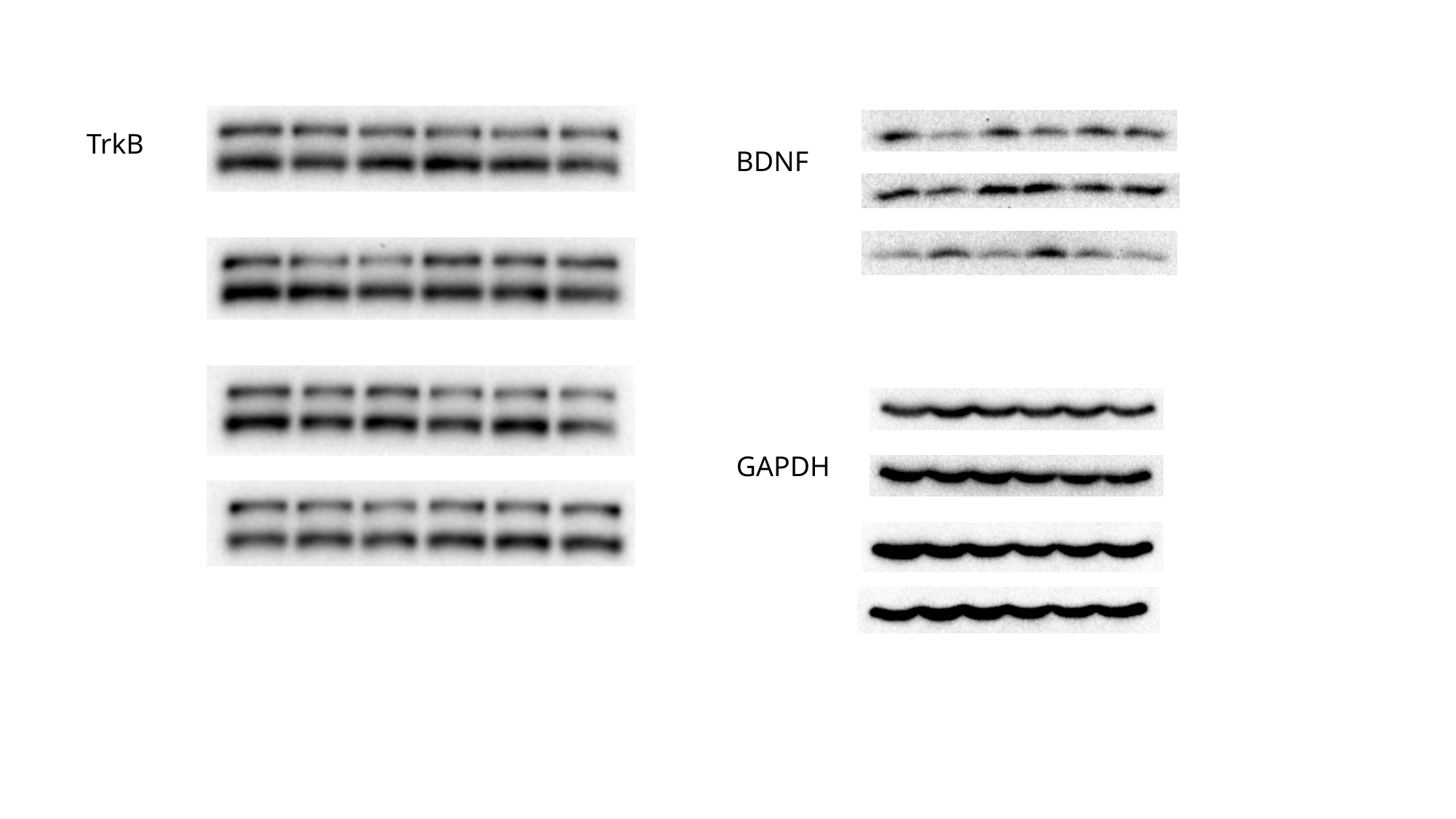

TrkB
BDNF
GAPDH

## Slide 6
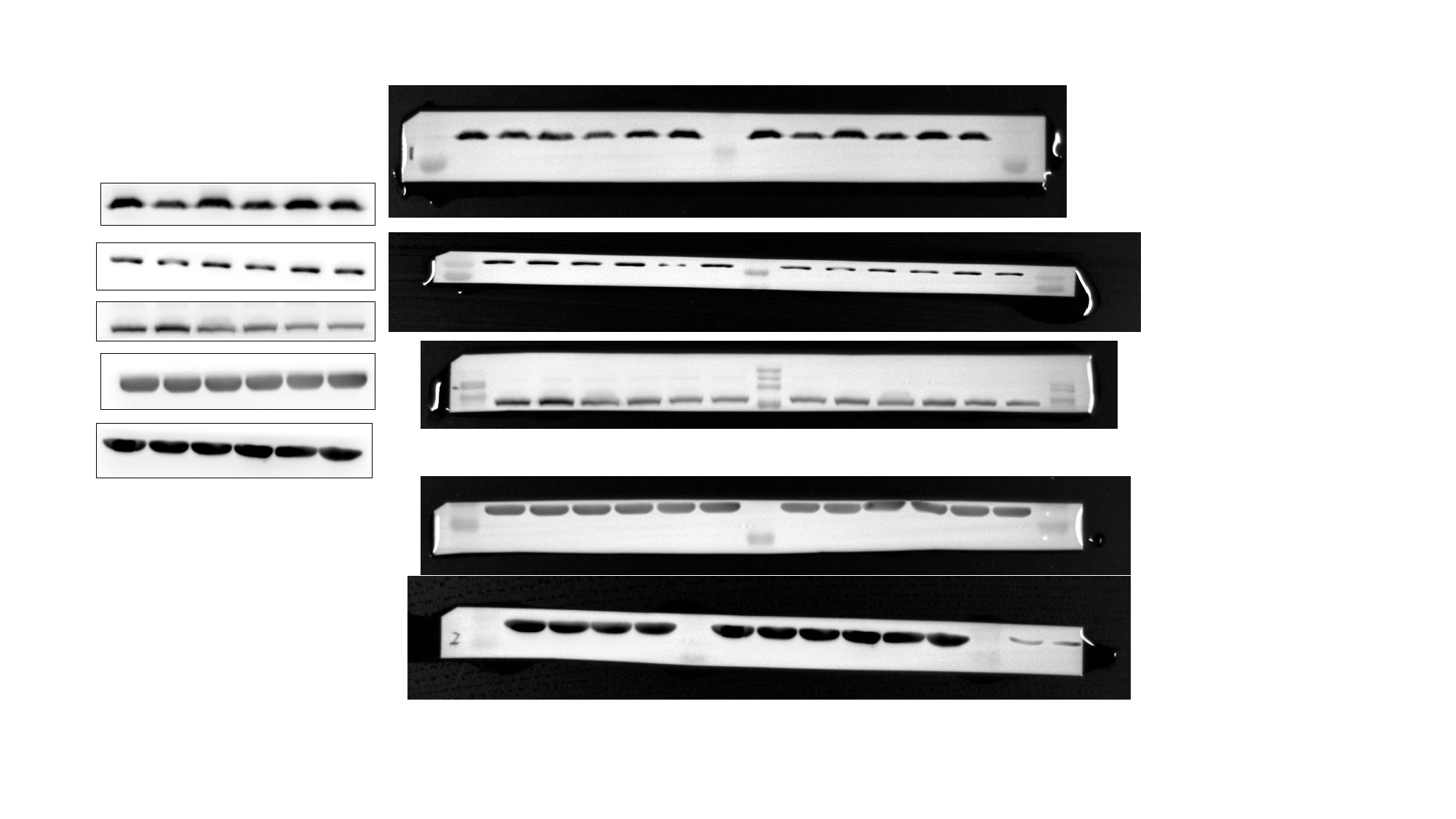

## Slide 7
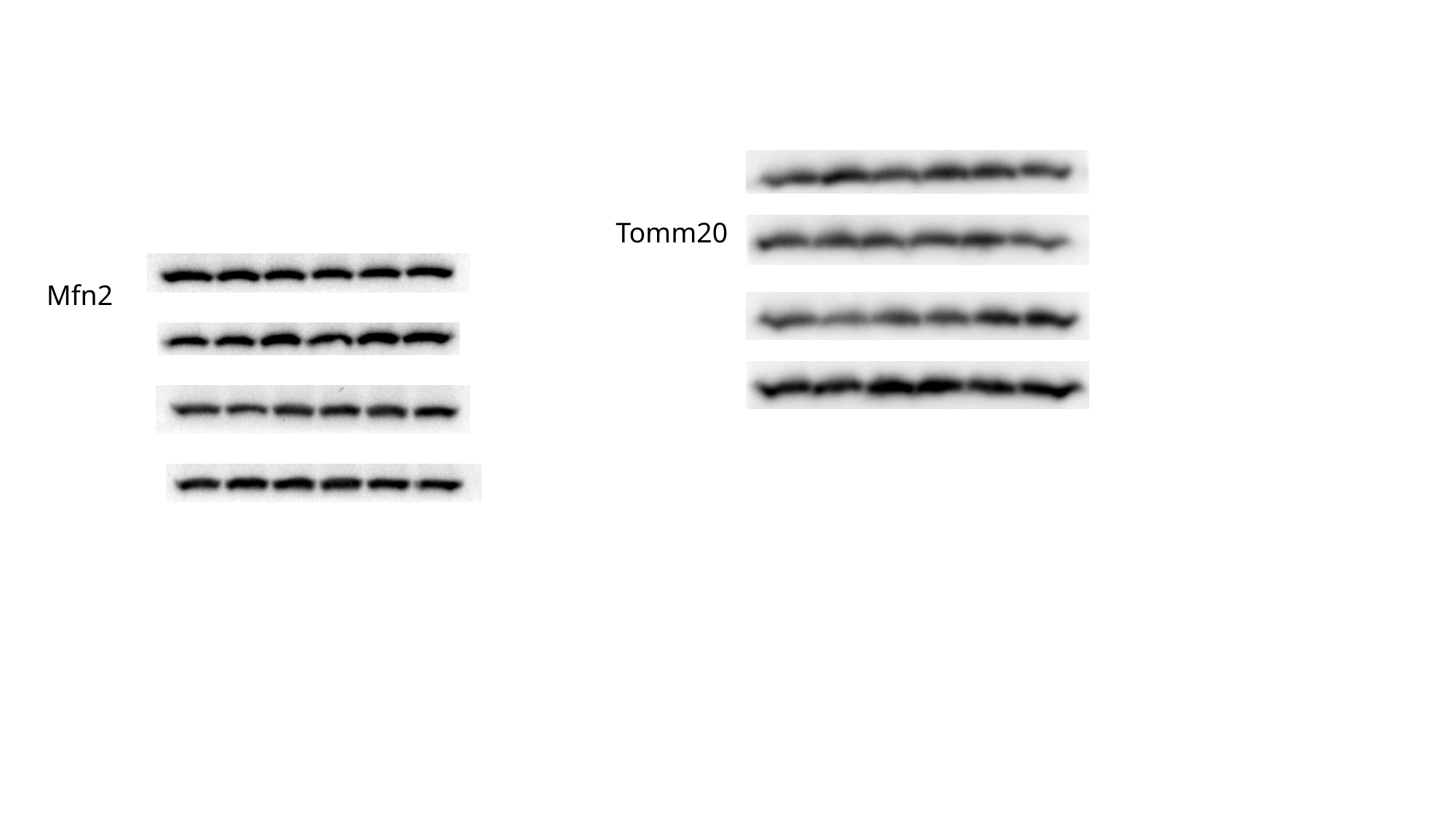

Tomm20
Mfn2
